# What Do We Know about Inequalities in NAFLD Distribution and Outcomes? A Scoping Review

**DOI:** 10.3390/jcm10215019

**Published:** 2021-10-28

**Authors:** Mar Talens, Natalia Tumas, Jeffrey V. Lazarus, Joan Benach, Juan M. Pericàs

**Affiliations:** 1 Research Group on Health Inequalities, Environment, and Employment Conditions, Department of Social and Political Science, Pompeu Fabra University, 08005 Barcelona, Spain; mar.talens01@estudiant.upf.edu (M.T.); natalia.tumas@upf.edu (N.T.); joan.benach@upf.edu (J.B.); 2 Public Policy Center (UPF-BSM), Johns Hopkins University-Pompeu Fabra University, 08005 Barcelona, Spain; 3 Centro de Investigaciones y Estudios sobre Cultura y Sociedad, Consejo Nacional de Investigaciones Científicas y Técnicas y Universidad Nacional de Córdoba, Córdoba 5016, Argentina; 4 Barcelona Institute for Global Health (ISGlobal), Hospital Clínic, University of Barcelona, 08036 Barcelona, Spain; jeffrey.lazarus@isglobal.org; 5 Transdisciplinary Research Group on Socioecological Transitions (GinTrans2), Universidad Autónoma de Madrid, 28049 Madrid, Spain; 6 Liver Unit, Internal Medicine Department, Vall d’Hebron University Hospital, Vall d’Hebron Institute for Research, 08035 Barcelona, Spain; 7 Centro de Investigación Biomédica en Red de Enfermedades Hepáticas y Digestivas (CIBERehd), 28029 Madrid, Spain

**Keywords:** non-alcoholic fatty liver disease, health inequalities, social determinants of health

## Abstract

With prevalence high and rising given the close relationship with obesity and diabetes mellitus, non-alcoholic fatty liver disease (NAFLD) is progressively becoming the most common chronic liver condition worldwide. However, little is known about the health inequalities in NAFLD distribution and outcomes. This review aims to analyze health inequalities in NAFLD distribution globally and to assess the health disparities in NAFLD-related outcomes. We conducted a scoping review of global health inequalities in NAFLD distribution and outcomes according to gender/sex, ethnicity/race, and socioeconomic position from PubMed’s inception to May 2021. Ultimately, 20 articles were included in the review, most (75%) of them carried out in the United States. Males were found to have a higher NAFLD prevalence (three articles), while available evidence suggests that women have an overall higher burden of advanced liver disease and complications (four articles), whereas they are less likely to be liver-transplanted once cirrhosis develops (one article). In the US, the Hispanic population had the highest NAFLD prevalence and poorer outcomes (seven articles), whereas Whites had fewer complications than other ethnicities (two articles). Patients with low socioeconomic status had higher NAFLD prevalence (four articles) and a higher likelihood of progression and complications (five articles). In conclusion, globally there is a lack of studies analyzing NAFLD prevalence and outcomes according to various axes of inequality through joint intersectional appraisals, and most studies included in our review were based on the US population. Available evidence suggests that NAFLD distribution and outcomes show large inequalities by social group. Further research on this issue is warranted.

## 1. Introduction

Non-alcoholic fatty liver disease (NAFLD) encompasses a spectrum of liver diseases ranging from simple hepatic steatosis (fatty liver) to steatohepatitis (NASH) to cirrhosis and hepatocellular carcinoma (HCC) [1]. NAFLD is currently considered the liver manifestation of metabolic syndrome (which also encompasses obesity, type 2 diabetes mellitus (T2DM), insulin resistance, and dyslipidemia) [2]. A significant part of the scientific community advocates for a change in terminology by which NAFLD is progressively replaced by metabolic-associated fatty liver disease (MAFLD) [3].

The increasing burden of disease owing to NAFLD and associated disorders has led to a massive scientific effort to better depict its epidemiology, natural history, and potential treatments. NAFLD has an estimated global prevalence of 25% of the general population, whereas the global prevalence of NASH has reached nearly 5% [4]. Therefore, NAFLD has become the most common cause of chronic liver diseases in many parts of the world [5], and NASH is one of the leading underlying causes of liver transplantation [6]. However, most countries, including Western countries, are not prepared to address NAFLD, lacking adequate policies and civil society awareness and engagement, guidelines, epidemiology, and care management [7]. Structural changes and new models of care are needed in order to improve NAFLD management and public health policies [7,8].

Besides the complex mechanisms underlying its pathogenesis [9], NAFLD is caused by interactions between many social, environmental, and genetic factors [10]. Among such factors, several social and economic conditions seem essential to understanding the current picture of NAFLD globally, as well as its differences between social groups. Although it is well known that social determinants of health (SDH) have a great impact on the non-communicable disease (NCDs) burden [11], there is an important gap in the literature of how they may be related to health inequalities in NAFLD global distribution and outcomes. Preliminary evidence from scattered studies suggests that Hispanic ethnicity [12] and male sex [13] are associated with a higher likelihood of having NAFLD in the United States. However, most studies have approached such disparities with a biomedical focus rather than a social lens, i.e., attributing them to the frequency of a polymorphism in the PNPLA3 gene in the Hispanic population [14], or to estrogen protection in premenopausal females [15].

This review aims to analyze the health inequalities on NAFLD distribution globally, including prevalence and incidence, and to assess the health disparities in NAFLD-related outcomes, including advanced liver disease and complications.

## 2. Materials and Methods

### 2.1. Selection of the Review Approach

A scoping review approach was chosen because this topic has not been extensively reviewed to date and has a complex and heterogeneous nature [16]. The methodologic proposal of Arksey and O’Malley was followed [17]. PRISMA guidelines were followed [18].

### 2.2. Literature Search Strategy

A PubMed search was conducted using the following keywords and expressions: [“NAFLD” OR “NASH” OR “fatty liver” OR “MAFLD”) AND/OR (“prevalence” AND/OR “distribution” AND/OR “incidence”, AND/OR “epidemiology”) AND/OR [“cirrhosis” AND/OR “hepatocellular carcinoma” OR “liver cancer”, AND/OR “advanced fibrosis” AND/OR “cardiovascular events” AND/OR “hepatic events”, AND/OR “mortality”] AND (“gender” AND/OR “sex” AND/OR “ethnicity” AND/OR “race” AND/OR “universal health coverage” AND/OR “socioeconomic position” AND/OR “socioeconomic status” AND/OR “education” AND/OR “housing”, AND/OR “income”) AND/OR [“disparities” OR “inequities” OR “equity” OR “inequalities”] from PubMed inception to 31 May 2021. Furthermore, a manual search of secondary references from screened articles was performed.

### 2.3. Eligibility Criteria

Articles were included if all the following inclusion criteria were met: (i) articles written in English; (ii) original articles; (iii) studies of at least one element of each of the two following pairs: NAFLD or MAFLD, distribution or outcomes (see definitions below); and iv) studies analyzing at least one axis of inequality (e.g., articles including SDH as subvariables for analysis without specific analysis of how they impact disparities in NAFLD distribution or outcomes were not included).

Articles were excluded if they: (i) mostly analyzed other causes of hepatic steatosis such as chronic viral hepatitis (C and/or B hepatitis viruses), drugs, or alcohol-related liver disease; (ii) did not exclude patients with elevated alcohol consumption (over 20 g/day in females and over 30 g/day in males), even if the most likely diagnosis was NAFLD; (iii) focused on pediatric population (<18 years); (iv) the study sample was smaller than 150 individuals; and (v) were reviews, meta-analyses, editorials, or commentaries.

### 2.4. Study Selection and Data Extraction

Articles were first screened based on the search keywords with title and abstract. Subsequently, a screening applying the inclusion/exclusion criteria was conducted, and ultimately a full-text reading was performed.

Extracted data included: (i) first author name, (ii) publication year, (iii) period of study, (iv) country of study, (v) study design and population, (vi) method of diagnosis, (vii) distribution index/analyzed outcomes, (viii) axes of inequality, (ix) results, (x) conclusions, and (xi) limitations of the study. Finally, data were summarized in two tables depending on whether the study analyzed NAFLD distribution (Table 1) or analyzed other outcomes such as advanced liver disease or complications (Table 2).

### 2.5. Definitions

The definitions that we used are as follows:

Advanced fibrosis: Liver fibrosis in stages 3 and 4 [5].

Advanced liver disease: NAFLD/NASH with advanced fibrosis or cirrhosis.

Axes of inequality: social determinants, such as social class, socioeconomic position, gender/sex, and ethnicity/race among, others that determine the opportunities for good health and reveal the existence of health inequalities due to hierarchies of power, prestige, or access to resources [19].

Cirrhosis: histological development of regenerative nodules surrounded by fibrous bands in response to chronic liver injury, which leads to portal hypertension and end-stage liver disease [20].

Complications of NAFLD: unfavorable results of the disease, comprising liver decompensation, liver cancer, liver transplant, cardiovascular events, hospitalization, and mortality.

Distribution of NAFLD: frequency and pattern of health events in the population, comprising prevalence and incidence.

Ethnicity/Race: historical social groups that share specific social, cultural, and some biological attributes [21].

Gender: a social construct regarding culture-bound conventions, roles, and behaviors for, as well as relationships between and among women and men and boys and girls, as opposed to sex, which is determined by the genetic background and secondary sexual characteristics [21].

Health inequalities: differences in health status or in the distribution of health resources between different population groups, which are not only unnecessary and avoidable, but in addition, are considered unfair and unjust [22].

Lean NAFLD/NASH: individuals with <25 BMI with clear evidence of NAFLD or NASH presence [23].

Liver decompensation: acute deterioration in liver function in a patient with cirrhosis and is characterized by jaundice, ascites, hepatic encephalopathy, hepatorenal syndrome, or variceal hemorrhage [24].

Outcomes of NAFLD: NAFLD-related consequences comprising NAFLD progression to advanced liver disease and NAFLD-related complications.

Social determinants of health: conditions in which people are born, grow, work, live, and age, and the wider set of forces and systems shaping the conditions of daily life. These forces and systems include economic policies and systems, development agendas, social norms, social policies, and political systems [25].

Social groups: sets of people whose ways of living have been historically determined by one or various social determinants of health such as gender, ethnicity, migration status, or socioeconomic position [25].

Social inequalities: in the context of health, health disparities, within and between countries, that are judged to be unfair, unjust, avoidable, and unnecessary (meaning: are neither inevitable nor irremediable) and that systematically burden populations rendered vulnerable by underlying social structures and political, economic, and legal institutions [21].

Socioeconomic position/status: an aggregate concept that includes both resource-based and prestige-based measures, as linked to both childhood and adult social class position. Resource-based measures refer to material and social resources and assets, including income, wealth, and educational credentials [21].

## 3. Results

### 3.1. Literature Search

**Figure 1:**
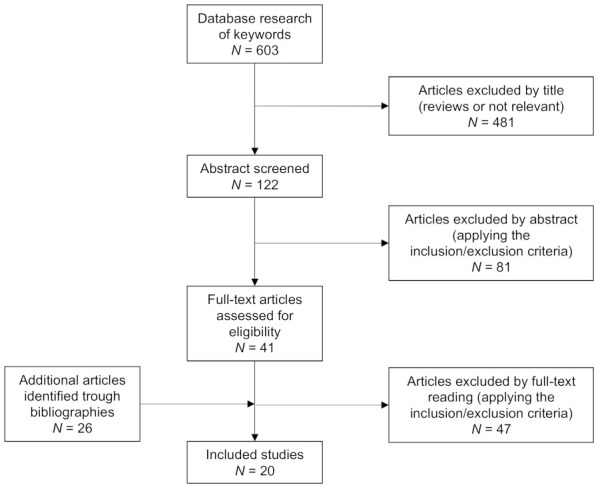
Flowchart of studies disposition throughout the search and article selection process according to PRISMA guidelines for scoping reviews [18].

Of 603 articles retrieved from the initial search, 481 (79.8%) were excluded by title, and 122 (20.2%) articles were selected for abstract screening. Of 122 articles, 81 (66.4%) were excluded applying the inclusion/exclusion criteria, and 41 (33.6%) were selected for full-text reading. At this point, 26 studies were added from reference lists of other selected articles. After the full-text reading, 47 did not meet the inclusion/exclusion criteria. Therefore, a total of 20 articles were selected to perform the review (Figure 1).

The studies were classified into two different groups—NAFLD distribution and NAFLD outcomes—although some of them were clustered in both groups (*n* = 4). The first group (Table 1) includes those articles addressing NAFLD distribution, i.e., analyzing the prevalence or incidence of NAFLD according to different axes of inequality (*n* = 17, 85%). The second group (Table 2) includes those articles that analyze NAFLD-related outcomes (*n* = 12, 60%), such as progression to advanced liver disease (*n* = 5, 25%) or complications (*n* = 8, 40%). Articles on advanced liver disease comprised advanced fibrosis (*n* = 2, 10%) and cirrhosis (*n* = 3, 15%), and complications included liver cancer (*n* = 3, 15%), hospitalization events (*n* = 1, 5%), liver transplants (*n* = 4, 20%), and mortality (*n* = 4, 20%). Yet, no study was found that analyzed health inequalities in cardiovascular events related to NAFLD or liver decompensation.

The most frequently addressed axes of inequalities were gender/sex (*n* = 16, 80%), ethnicity (*n* = 10, 50%), and socioeconomic position (*n* = 7, 35%). Although 12 studies analyzed more than one axis of inequality (60%), only 4 articles (20%) analyzed the intersection between them.

### 3.2. NAFLD Distribution

#### 3.2.1. Gender/Sex

In the general population, NAFLD is more prevalent among men than women [26,27,28]. Specifically, in China, there is an NAFLD prevalence of 26.5% in men and 20.1% in women [27], and among US citizens, men comprise 58% of the total population with NAFLD [26]. However, this prevalence disparity is not conserved when models are adjusted for body mass index and age. Among lean or non-overweight populations, women seem to have a higher prevalence than men [23], while when only postmenopausal women are taken into account, NAFLD prevalence seems to be the same between males and females [28,29]. Additionally, men tend to have an NAFLD onset sooner than women. Younger men (≤45 years) have a higher prevalence than older men (30% vs. 24%) [27]. Conversely, women have a higher prevalence at an older age. In the Chinese population, women aged less than 45 years have a lower prevalence of NAFLD compared to women aged more than 45 years (15% vs. 22.8%) [27], and in the US population, elderly females are more likely to have NAFLD than younger females (28–14%) [37]. Further, it has also been described that increased age is associated with a decreased risk of having NAFLD in men (OR 0.87) and increased risk in women (OR 1.22) [27].

Therefore, although NAFLD seems to affect more males than females, age and menopausal status play an important role in sex inequalities in NAFLD prevalence. Moreover, even though several studies refer to this SDH as gender, the analyses are based on biological sex (male vs. female) only.

#### 3.2.2. Ethnicity/Race

The exact prevalence of ethnicity differs according to the methodology employed and the population investigated. Overall, the prevalence of NAFLD appears to be higher among Hispanics, followed by non-Hispanic Whites and Asians, and lastly, African Americans in the US population [26,29,30,31]. Furthermore, this ethnic disparity has also been observed in hospitalized patients [32] and lean-NAFLD patients [23].

In the US population, NAFLD prevalence is higher in Mexican-Americans (21.2%) followed by non-Hispanic Whites (12.5%), and lower in African Americans (11.6%) [30]. Even so, a recent prospective study suggests that NAFLD prevalence among the Hispanic population might be much higher (40%) [28,29]. Additionally, Hispanic ethnicity is a risk factor to develop NAFLD [23,33], whereas African Americans have lower odds to develop fatty liver disease (OR 2.03 vs. 0.42) [33].

In addition, there are substantial differences in the mean age of NAFLD onset by race. Although it seems that Mexican Americans have an earlier NAFLD onset (36.7 years) [30], it is not clear whether African Americans or non-Hispanic Whites have the latest onset. Schneider et al. observed that the mean age for Non-Hispanic Whites is 43.1 years, while the mean age for African Americans is 39.5 years [30]. Another study suggested that African Americans with NAFLD were significantly older than other racial or ethnic groups [31].

Although Hispanic ethnicity has the highest NAFLD prevalence, Japanese Americans have a greater susceptibility to intra-abdominal adiposity, which is a risk factor for NAFLD [28]. Even though Hispanics have a higher visceral fat mass, once the total mass is accounted for, their visceral fat mass proportion is similar to that of Whites [28]. There are also differences in liver enzymes such as aspartate and alanine transaminases levels, which are used as NAFLD biomarkers. Although AST and ALT levels are the highest among Hispanics, African Americans have significantly higher mean AST levels compared to non-Hispanic Whites [30]. Hence, although the adiposity body distribution and AST/ALT levels differ between ethnicities, they do not follow the same prevalence pattern as NAFLD.

Hispanics appeared to have a higher NAFLD prevalence, a sooner onset, and a worse metabolic profile than other ethnicities. Of note, all the studies that analyze ethnic disparities were performed among the US population.

#### 3.2.3. Socioeconomic Position/Status

Overall, NAFLD prevalence seems to be higher among individuals with lower socioeconomic position in Western countries [33,34]. However, data coming from Eastern countries is not so consistent [35,36]. A study conducted in South Korea found that people with a low socioeconomic status have a significantly higher risk of developing NAFLD (OR 1.7) [36]. A Chinese study also found that people with a higher median income had a 1.96 higher risk of developing NAFLD than the low-income population [35]. However, this study had a notable selection bias that limits external validity [35].

In the field of NAFLD, the socioeconomic position has been particularly studied by addressing how it relates to food insecurity and the limited or uncertain access to nutritionally adequate and safe foods [33,34]. Approximately 29% of US adults in low-income households with NAFLD live in food-insecure households [33]. Subjects who live under food insecurity have higher odds of developing NAFLD (OR 1.38) [33]. Similarly, in the Iranian population, the prevalence of food insecurity is much higher among the NAFLD population (56.8%) than among individuals without NAFLD (26.1%) [34].

### 3.3. NAFLD Outcomes

#### 3.3.1. Advanced Liver Disease in NAFLD

##### Gender/Sex

Although a global population study concluded that among the population below 70 years, males had a higher prevalence of cirrhosis and several DALYs (Disability-Adjusted Life Years) because of liver disease [38]. In the US, women seem to have a higher prevalence of NASH-related cirrhosis than males, both compensated and decompensated [32,39]. Little has been studied about gender/sex differences in NAFLD progression to advanced fibrosis; however, it seems that the prevalence of advanced fibrosis is similar (OR 1.6 vs. 1.4) when only postmenopausal women are analyzed [40]. Furthermore, among females, the elderly have higher rates of advanced fibrosis at NAFLD onset (OR 1.8) [40].

##### Ethnicity/Race

No studies analyzing the impact of ethnicity/race as a risk factor of either advanced liver fibrosis or cirrhosis nor the comparative risks or burdens in NAFLD patients were found.

##### Socioeconomic Position/Status

Advanced liver disease is more prevalent in individuals with lower socioeconomic status [33,36,38]. In geographic regions with an overall lower socioeconomic position, there is higher cirrhosis prevalence [38]. Furthermore, among low-income households, food insecurity is associated with higher odds of advanced fibrosis (OR 2.20) [33].

**Table 2:** Summary of included studies assessing NAFLD outcomes.

Author, Year	Period Country	Design and population	Diagnosis method	Outcomes	Axes of Inequality	Results	Conclusions	Limitations
Noureddin, 2018 [6]	2004–2016 US	Study cohort logistic Regression DB: UNOS/OPTN *n* = 127,164 Age ≥ 18	Not specified	COMP (LT) and AdLD (NASH)	Sex Ethnicity	NASH was the leading cause of LT for women and the 2nd leading cause for men (following alcoholic liver disease). NASH increased as the cause in all ethnic subgroups and was the leading cause in 2016 among Asian, Hispanic, and non-Hispanic white females.	NASH is the second leading cause for LT waitlist registration/LT and in females, the leading cause. NASH will rise to become the leading indication for LT in males	Includes other liver diseases.
Younossi, 2012 [23]	1988–1994 US	Cross-sectional study DB: NHANES III *n* = 11,613 Age ≥ 20	Ultrasound	AdLD (NASH)	Ethnicity	NASH is independently associated with being Hispanic (OR 1.72; 95% CI 1.28–2.33) and less associated with being African American (OR 0.52, 95% CI 0.34–0.78).	Patients with NASH are commonly Hispanic and less likely to be African American.	Does not perform an intersectional analysis.
Williams, 2011 [26]	2007–2010 US	Prospective Brooke army medical center *n* = 328 (156 positive US) Age 28–70	Ultrasound and optional liver biopsy	AdLD (NASH)	Ethnicity Sex	NASH prevalence: Hispanics 19.4% > Caucasians 9.8% (*p* = 0.03) NASH has a prevalence of 22.2% in the diabetic population. NASH patients are predominantly male (65%).	NASH prevalence is higher than estimated. Hispanics and patients with diabetes are at the greatest risk for NASH.	Does not perform an intersectional analysis.
Wu, 2018 [37]	1993–2017 US	Retrospective analysis Medical records. *n* = 1206 Age ≥ 18	Histologically (biopsy at surgery), imaging (CT and MRI) and AFP	COMP (HCC)	Sex	Females develop HCC at a significantly older age (66 years vs. 61.6 years, *p* < 0.001). Elderly females (≥65 years) are more likely than elderly males to have NAFLD/NASH-related HCC (28.0% vs. 14.8%, *p* = 0.0006).	Older females with HCC have more NAFLD/NASH and may be overlooked by current surveillance guidelines.	Includes other liver diseases. Does not perform an intersectional analysis.
Adejumo, 2019 [32]	2007–2014 US	Retrospective cross-sectional study DB: HCUP-NIS *n* = 210,660 Age	ICD-9 NIS does not contain information on how NAFLD was diagnosed	COMP (hospitalization and mortality) AdLD (cirrhosis)	Sex Ethnicity SES	Females had a 5% shorter LOS than males (4.35 [95% CI, 4.26–4.45] vs. 4.18 [95% CI 4.08–4.27]; *p* < 0.0001) and 10% lower odds of mortality (AOR 0.91, 95% CI 0.83–0.99; *p* = 0.03). African American had a 7% longer LOS than Whites (4.48 [95% CI 4.34–4.62] vs. 4.20 [95% CI 4.11–4.29] days; *p* < 0.0001); and 14% greater odds of unfavorable discharge compared with Whites (AOR 1.14, 95% CI 1.06–1.22; *p* < 0.0001). Non-private insured patients had 14% higher mortality than those with private insurance (AOR 1.14, 95% CI 1.09–1.67; *p* = 0.002). Females had a higher frequency of compensated (2.39% vs. 1.93%) and decompensated (2.80% vs. 2.31%) cirrhosis.	Males, Hispanics, individuals with non-private health insurance are disproportionately affected, with higher NAFLD complications.	Does not perform an intersectional analysis.
Golovaty, 2020 [33]	2005–2014 US	Cross-sectional analysis DB: NHANES *n* = 2627 of low-income adults Age ≥ 20	NAFLD fibrosis score (NFS) for advanced liver fibrosis	AdLD (advanced fibrosis)	SES (Food Insecurity)	A total of 29% (95% CI: 26%, 32%) were food-insecure. Multivariable model: food-insecure adults are more likely to have advanced (adjusted OR 2.20; 95% CI: 1.27–3.82).	Food insecurity may be independently associated with NAFLD and advanced fibrosis among low-income adults in the US.	Does not perform an intersectional analysis.
Zhang, 2021 [38]	1990–2017 Global	Retrospective analysis DB: GBD study, 2017	Not specified	AdLD (NASH, cirrhosis)COMP (liver cancer)	Sex SES	The global DALYs numbers of liver cancer due to NASH were negatively associated with SDI levels (r = −0.409, *p* < 0.001). The global prevalence number of liver cancer due to NASH peaked at 60–64 years in males and 65–69 years in females. Globally, the burden was heavier in males compared with females. In groups > 70 years, the DALYs in females for cirrhosis started to be higher than in males.	The global burden of NASH-associated liver cancer has increased significantly since 1990, with age, sex, and geographic disparity.	The accuracy of the GBD estimates was limited by the quality and availability of original hospital and claims data.
Mazumder, 2020 [39]	2006–2012 US	Retrospective analysis DB: OPTN *n* = 20,045 Age ≥ 18	Not specified	COMP (mortality, LT) AdLD (NASH, cirrhosis)	Sex	Females had higher rates of NASH (29.8% vs. 21.2%, *p* < 0.001) than males. Although lower rates of listing (7.5% vs. 9.8%; *p* < 0.001) and LT (3.5% vs. 5.2%; *p* < 0.001) among women, no significant difference was noted for either all-cause mortality (sHR 1.09; 95% CI 0.88–1.35) or liver-related death (sHR 1.12; 95% CI 0.87–1.43). Females had higher rates of NASH-related cirrhosis (29.8% vs. 21.2% *p* < 0.001)	In patients with cirrhosis, women were not at an increased risk of liver-related death despite lower rates of listing and transplantation.	Does not perform an intersectional analysis.
Yang, 2014 [40]	2007–2010 US	Cross-sectional study DB: DUHS NAFLD Clinical DB *n* = 203 Age ≥ 18	Liver biopsy	AdLD (advanced fibrosis)	Sex Ethnicity	The likelihood for greater fibrosis severity was ACOR: 1.4 (95% CI 0.9–2.1, *p* = 0.17) for PMP women and ACOR 1.6 for men (95% CI 1.0–2.5, *p* = 0.03), with premenopausal women as a reference. Before age 50, men had a higher risk of having greater severity of fibrosis than women ACOR 1.8 (95% CI, 1.1–2.9, *p* = 0.02). After age 50, the protective effect observed in women appeared to be eliminated (ACOR 1.2, 95% CI 0.7–2.1, *p* = 0.59). Age ≥ 50 was associated with an increased risk of having more advanced fibrosis only among women (ACOR 1.8, 95% CI 1.2–2.7, *p* < 0.01)	Men are at a higher risk of having more severe fibrosis compared to women before menopause, while postmenopausal women have a similar severity of liver fibrosis compared to men.	Does not perform an intersectional analysis. Purely biological approach.
Phipps, 2020 [41]	2000–2014 US	Multicenter retrospective study Hospital centers *n* = 5327Age ≥ 18	Imaging or biopsy	COMP (HCC)	Sex	Among patients with HCC, NAFLD was the underlying etiology of liver disease in 23.3% of cases compared with 12.4% of men.	The frequency of underlying NAFLD was significantly higher in women than men.	They do not classify women as pre/PMP. Includes other liver diseases.
Loy, 2018 [42]	2005–2012 US	Retrospective study with an 8-year follow-up DB UNOS/STAR *n* = 76,149	Not specified	COMP (LT and mortality)	Sex	NASH is a more frequent indication for LT listing in women than men. LT is lower among women than men with NASH (52.4% vs. 64.3%), and women are more likely to experience death on the WL (17.1% vs. 11.4%). In multivariable analysis adjusting for covariates, the rate of LT remained lower for women with NASH (aHR 0.81 95% CI 0.75–0.88).	Women with NASH cirrhosis had a higher risk of death on the LT waiting list and were less likely to receive LT compared to men.	Does not perform an intersectional analysis.
Ochoa, 2020 [43]	2002–2013 US	Retrospective analysis DB: UNOS/OPTN *n* = 45,767 Age ≥ 18	Not specified	COMP (mortality, LT)	Ethnicity	Non-Hispanic whites are more commonly transplanted for NASH than Hispanics. Hispanics had a decreased risk of death when transplanted for NASH (HR 0.84, 95% CI 0.71–0.99; *p* = 0.04).	Hispanics have similar or better long-term post-LT outcomes compared to non-Hispanic whites despite a worse pretransplant risk factor profile.	Only analyzes Hispanics and non-Hispanic Whites.

Footnote: ACOR (adjusted cumulative odds ratio), AFP (alpha-fetoprotein), aHR (adjusted hazard ratio), AdLD (advanced liver disease), AOR (adjusted odds ratio), CI (confidence interval), COMP (complications), CT (computed tomography), DALYs (disability-adjusted life years), DB (database), DUHS (Duke University Health System), GBD (global burden of disease), HCC (hepatocellular carcinoma), HCUP (Healthcare Cost and Utilization Project), HR (hazard ratio), ICD-9 (international classification of disease), LOS (length of stay), LT (liver transplant), M (men), MRI (magnetic resonance imaging), NAFLD (non-alcoholic fatty liver disease), NASH (non-alcoholic steatohepatitis), NIS (national inpatient survey), NHANES (national health and nutrition examination survey), OPTN (Organ Procurement and Transplantation Network), OR (odds ratio), PMP (postmenopausal), RF (risk factor), SDI (sociodemographic index), SES (socioeconomic status/position), sHR (subdistribution hazard ratio), STAR (Standard Transplant Analysis and Research), UNOS (United Network for Organ Sharing), W (women), WL (waitlist).

#### 3.3.2. NAFLD-Related Complications

##### Gender/Sex

Regarding liver cancer, in a Chinese cohort of 1206 patients with HCC, Wu et al. found that just 25% were women, who were more likely to undergo HCC surveillance, had smaller tumor size at diagnosis, and less vascular involvement [30]. However, 21.5% of females had NAFLD as underlying liver disease, whereas only 7.2% of males did. Moreover, males who met the Milan criteria for liver transplantation were more likely to undergo liver transplant than women who met the criteria [30]. In an American cohort including 5327 patients with HCC, 22.6% were women, who had a significantly higher frequency of NAFLD (23% vs. 12%) and lower frequency of alcoholic liver disease (5% vs. 15%). The proportion of noncirrhotic HCC was higher among women (17% vs. 10%). Women had less-advanced HCC at presentation by tumor, node, metastasis staging, a higher proportion within Milan criteria (39% vs. 35%), and a greater overall survival (2.5 ± 2.9 years vs. 2.2 ± 2.7 years) [41].

NAFLD-related mortality according to sex differs across studies. Adejumo and colleagues found that in the US from 2007 to 2014 women had a significantly lower in-hospital mortality [32]. However, this study was based on ICD-9 diagnosis code, whose ability to capture NAFLD-related diagnosis and complications is severely limited. Meanwhile, in a population of 76,149 patients listed for liver transplant between 2005 and 2012 of whom 7.2% were listed for NASH, Loy et al. found that women with NASH cirrhosis had a higher risk of death on the liver transplant waiting list [42].

NASH is the main cause of liver transplant among females and the second one in males, right after alcoholic liver disease [6,42], yet the US women with NASH are 19% less likely to receive a liver transplant compared to men [42].

##### Ethnicity/Race

NASH has increased as a cause of liver transplant in all ethnic subgroups. Ethnic inequalities have been observed in NAFLD-related complications such as liver transplant and hospitalization events. In 2016, NASH was the leading liver transplant cause among Asian, Hispanic, and non-Hispanic White females [6]. According to the lowest NASH prevalence, African American population had a lower increase in the rate of waitlist registration for NASH compared to the other ethnic groups [6]. Additionally, although the highest NASH prevalence has been seen among the Hispanic population [23,26], non-Hispanic White patients are more commonly transplanted for NASH compared to Hispanics in the US [43]. Although seemingly the lowest NASH prevalence is found among the African American population [23,26], these have a 14% greater odds of unfavorable discharge compared with whites [32].

##### Socioeconomic Position/Status

Patients with low socioeconomic status have higher liver cancer rates [31]. The odds of dying on the waitlist increase by 14% in NASH patients without health insurance or self-paid [32].

## 4. Discussion

NAFLD distribution and outcomes show large inequalities by social group. Males have higher NAFLD prevalence, while available evidence suggests that women have an overall higher risk of progression to advanced liver disease and more complications, whereas they are less likely to undergo transplantation once cirrhosis develops. In the US, the Hispanic population has the highest NAFLD prevalence and poorer outcomes, whereas Whites have fewer complications than other ethnicities. Patients with low socioeconomic status have higher NAFLD prevalence and higher likelihood of progression and complications. Globally, there is a lack of studies analyzing NAFLD prevalence and outcomes according to various axes of inequality through joint intersectional appraisals, and most studies included in our review are based on the US population.

Although in the general population males have a higher prevalence, if only postmenopausal women are taken into account, the prevalence is similar between male and female individuals. In addition, females have a greater risk of progression to advanced liver disease. In a systematic review, Balakrishnan et al. found that although they have a lower risk of NAFLD than men, once NAFLD is established, women have a higher risk of advanced fibrosis, especially after age 50 [13]. Although estrogens have been shown to play an important role as a protective factor against steatosis and therefore may at least partially account for an earlier NAFLD onset among men [15], little research has been carried out to elucidate such sex differences in NAFLD prevalence. Because females have lower healthcare access [44], they might be underdiagnosed or may receive a later diagnosis that can lead to further progression of the disease. Moreover, the fact that males tend to have a higher alcohol consumption than females [31] might predispose healthcare providers to rule out liver disease in men rather than women. Thus, aspects such as access to healthcare, living and working conditions, and gender roles should be included in further research exploring gender disparities in NAFLD.

Ethnicity/race is the axis of inequality that has been more largely documented (50% of analyzed studies addressed it), and it has been shown to have a greater influence on health inequalities in NAFLD prevalence and outcomes. In particular, the Hispanic population has a higher NAFLD prevalence and burden of advanced liver disease and complications. Yet, ethnic disparities in NAFLD distribution have mostly been studied among the US population [23,26,29,30,31], and further studies should be conducted to determine if these inequalities are conserved in other countries. Ethnic disparities are not fully explained by the metabolic profile and the cardiometabolic comorbidities of the individuals, such as obesity, T2DM, or hypertension. For instance, although African Americans have the lowest prevalence, they have a worse cardiovascular profile [29] and similar diabetes and obesity prevalence as for other ethnicities [45,46]. Most studies suggest that ethnic disparities might be better explained by PNPLA3 polymorphisms, which are more frequent in the Hispanic population and promote fat accumulation on the liver [14]. Although genetic polymorphisms and metabolic profiles may play an important role in ethnic disparities, living and working conditions have a major impact on health inequalities beyond individual factors. The higher NAFLD prevalence among Hispanic ethnicity could be also explained by the fact that they have the worst health care access [47,48] and health insurance coverage [49]. Hence, further research is needed to understand the gene–environment interactions and epigenetic mechanisms including environmental and social factors determining NAFLD onset and progression.

Socioeconomic status is tightly associated with the risk of developing NAFLD. Our findings are consistent with previous studies that demonstrate that NCDs are more prevalent among the population with lower socioeconomic status [50]. Additionally, food insecurity seems to play an important role in NAFLD onset, as food-insecure households have been described as having a greater burden of cardiometabolic diseases, such as diabetes, obesity, and hypertension [51,52]. Food insecurity is characterized by difficult access to healthy food, which can lead to an early obesity onset in young ages that ends up with a metabolic syndrome. Therefore, since NAFLD is a metabolic disease, the increased NAFLD prevalence in the food-insecure population can be related to a higher prevalence of metabolic syndrome among this population [33]. In addition, low socioeconomic position is also associated with other individual outcomes such as depression and anxiety, or community conditions such as poor healthcare access or absence of safe spaces to exercise [51] that could also participate in inequalities in NAFLD distribution and outcomes.

NAFLD, like all non-communicable diseases, is a multifactorial disease that is influenced by both individual factors, such as behavior or genetics, and contextual factors such as culture, demographics, income inequality, environment, or health policies [51,53]. Nevertheless, most studies are only focused on the individual level, emphasizing self-responsibility through a “victim-blaming” approach and ignoring the deeper social, economic, and environmental determinants of living and working conditions that can lead to NAFLD onset [54]. Even though genetic and metabolic factors have a paramount impact on NAFLD prevalence and progression, they do not explain all the observed inequalities. In the field of hepatology, social conditions are closely linked to liver disease prevalence and outcomes, as chronic liver disease disproportionately impacts racial and ethnic minorities, low socioeconomic position communities, and immigrants [55]. Therefore, NAFLD should be understood as context-sensitive and analyzed by taking into account the jointly influence of individual factors, such as genetics or behaviors, social factors such as culture, race, income, and education; environmental factors, such as availability and access to healthy foods, energy-dense foods or the proximity of safe walking spaces; and macro-level factors such as the influence of media and advertising, access to health care or governmental policies [51]. Hence, it is urgent to study NAFLD, as well as all NCDs, through approaches embedding multilevel analysis that allows examining simultaneously both community-level and individual-level SDH that can influence NAFLD onset and progression [56].

It should be noted that besides the scarcity of studies paying attention to SDH and inequalities in the field of NAFLD, there are two other relevant barriers that hinder an accurate epidemiological approach. Although the definition of NAFLD relies on the presence of steatosis in at least 5% of hepatocytes, in reality, the approach to NAFLD diagnosis widely varies across studies, which largely impacts the reports of NAFLD prevalence. Moreover, whereas the concept of MAFLD has been proposed by some experts to facilitate the clinical approach to fatty liver disease in patients with relevant metabolic disorders [57], it is a potential source of heterogeneity both in clinical and epidemiological studies. By neither excluding patients with alcohol consumption over the dose traditionally considered for risk for liver injury nor those with concomitant liver disease, MAFLD widely differs from NAFLD. Hence, data from studies reporting on MAFLD distribution and outcomes should be carefully analyzed, and direct extrapolation between MAFLD and NAFLD should be avoided.

This study has several limitations. First, this is the first review on a vast topic and it only explored the PubMed database, and only articles written in English were included, so some articles may have been missed. Yet, scoping reviews are a rigorous, transparent, and replicable method that provide a robust assessment of the potential size and scope of available research literature and allow for identifying knowledge gaps when there is not enough evidence in the field to raise a specific question that can be studied through a systematic review [16]. Second, most studies addressing advanced liver disease in NAFLD also include other causes of liver disease, such as viral hepatitis or alcoholic liver disease. Thus, it is difficult to extract data that include only NAFLD/NASH as a cause of liver disease. Third, we have not analyzed some axes of inequalities that can have an important influence on health disparities, such as migration status or other axes. Fourth, most of the included studies do not specify how gender has been described and may be referring to biological sex as gender. However, we acknowledge that part of the differences between men and women on NAFLD are related to gendered social roles and not to biological features. Additionally, although gender is a spectrum of many genders, we have only analyzed male and female identities, as they are the most studied ones. Fifth, most studies included in our review concern the US population, therefore limiting the external validity; e.g., some geographical settings such as South America and some Asian countries appear to have higher burdens of NAFLD [4], but data are lacking on its distribution according to SDH. Fifth, we decided to include only original articles and therefore excluded both meta-analyses and reviews from our study, which might have led to the omission of relevant studies addressing at least one of the axes of inequality in the distribution and outcomes of NAFLD [58]. Finally, the included studies do not specifically analyze the impact of social determinants of health on the onset of health inequalities, and most of them do not assess the intersection of more than one axis of inequality. Hence, more research should be conducted to understand how the interaction between different social determinants of health (e.g., employment and neighborhood environment) leads to social health inequalities in NAFLD.

## 5. Conclusions

Hispanics, males, and people with low socioeconomic position present higher rates of NAFLD, whereas females present relatively poorer outcomes. Overall, little has been studied on the relationship between SDH and NAFLD. Moreover, most research has been carried out in the US; hence, evidence to provide an accurate assessment of the relationship between SDH and NAFLD in many regions is lacking. In addition, intersectional approaches encompassing several SDH and axes of inequality that provide an integrated account of both the biomedical and social determinants of NAFLD are needed. Further research is warranted to shed light on the impact of SDH on NAFLD distribution and outcomes both at the national and global levels.

## Figures and Tables

**Table 1:** Summary of included studies assessing NAFLD distribution.

Author	Period, Country	Design and Population	Diagnostic Methods	Distribution Index	Axes of Inequality	Results	Conclusions	Limitations
Younossi, 2012 [23]	1988–1994 US	Cross-sectional study DB: NHANES III *n* = 11,613Age ≥ 20	Ultrasound	Prevalence	Sex	Lean NAFLD is independently associated with younger age, female sex, and a decreased likelihood of having IR and hypercholesterolemia (*p* < 0.05)	Lean individuals with NAFLD have a different clinical profile than overweight-obese individuals with NAFLD.	Does not perform an intersectional analysis.
Williams, 2011 [26]	2007–2010 US	Prospective Brooke Army Medical Center *n* = 328 (156 positive ultrasound) Age 28–70	Ultrasound and optional liver biopsy	Prevalence	Ethnicity Sex	NAFLD prevalence: Hispanics (58.3%) > Caucasians (44.4%) > African Americans (35.1%) Males (58.9%) > Females (41.1%) A 74% of the diabetic population was diagnosed with NAFLD.	NAFLD prevalence is higher than the estimate. Hispanics and patients with diabetes are at the greatest risk for NAFLD.	Does not perform an intersectional analysis.
Zhou, 2019 [27]	2012–2013 China	Cross-sectional study *n* = 4002 Age 25–75	Abdominal computed tomography (Liver-spleen ratio ≤ 1.1)	Prevalence	Sex SES	NAFLD prevalence: 26.5% of men > 20.1% women. NAFLD prevalence was highest in younger men and older women. Younger men aged ≤45 years have a higher prevalence than older men aged >45 years (30.1% vs. 24.2%, *p* = 0.021), whereas younger women aged ≤45 years have a lower prevalence of NAFLD compared to older women aged >45 years (15.2% vs. 22.8%, *p* < 0.001). A higher proportion of men with NAFLD had an annual household income ≥ 75000CNY.	NAFLD is common in a gentrifying Chinese population, particularly in younger men of high socioeconomic status and older women with sedentary behavior who eat red meat.	The studied region may not be representative of all of China.
Lim, 2019 [28]	2013–2016 US	Cross-sectional study. DB: Multiethnic cohort study *n* = 1794 Age 58–74 (PMP women)	Abdominal MRI scan	Prevalence	Ethnicity Sex	NAFLD prevalence: Latinos (56% M vs. 47% W) > Japanese Americans (38% M vs. 46% W) > Native Hawaiians (35%M vs. 42%W) > Whites (23% M vs. 21% W) > African Americans (21%M vs. 18% W). Compared with African Americans, Japanese Americans had a greater mean visceral fat area (45%M vs. 73%W), and mean liver fat (61%M vs. 122%W)	The results suggest strong evidence for disproportionately greater susceptibility to intra-abdominal adiposity among Japanese compared to other ethnic/racial groups and greater prevalence of NAFLD in Latinos.	Purely biological approach
Remigio-Baker, 2017 [29]	2002–2005 US	Retrospective study DB: MESA *n* = 326 Age 45–84 (PMP women)	Computed tomography	Prevalence	Ethnicity Sex	The prevalence of NAFLD was similar for both women and men; highest among Hispanics (40.1%) and lowest among Blacks (11.1%) and Chinese (11.6%). Among Blacks, NAFLD was associated with a greater AAC prevalence (41%), and among women, NAFLD was associated with a 13% greater prevalence of AAC.	NAFLD is related to the presence of AAC on Blacks and women. These suggest disparities in the pathophysiologic pathways in which atherosclerosis develops.	Does not perform an intersectional analysis.
Schneider, 2014 [30]	1988–1994 US	Cross-sectional study DB: NHANES III *n* = 9675 Age 20–74	Ultrasound and elevated AST	Prevalence	Ethnicity Sex	NAFLD prevalence: Mexican-Americans (21.2%) > non-Hispanic Whites (12.5%) > African American (11.6%). Mexican-Americans were more likely to have NAFLD (OR 1.67, 95% CI 1.26–2.22). African Americans were significantly less likely to have NAFLD with elevated aminotransferases (OR 0.51, 95% CI 0.27–0.97). Racial differences were attenuated among those with normal BMI.	Mexican-Americans had a significantly higher prevalence of NAFLD. Racial differences are not fully explained by lifestyle, adiposity, and metabolic factors.	Does not perform an intersectional analysis.
Weston, 2005 [31]	1998–2000 US	Cross-sectional study DB: Chronic Liver Disease Surveillance Study *n* = 742 (333 with NAFLD) Age 20–74	Radiology and an optional liver biopsy	Prevalence	Sex Ethnicity	Among people with NAFLD, the majority were nonwhite: Hispanics (28%), Asians (18%), African Americans (3%), and other race(s) (6%). African Americans with NAFLD were significantly older than other racial or ethnic groups (*p* < 0.001), and in Asians, NAFLD was 3.5 times more common in males than in females (*p* = 0.016). Hispanics with NAFLD were over-represented (28–10%) and whites were under-represented (45–59%).	These racial and gender variations may reflect differences in genetic susceptibility to visceral adiposity, including hepatic involvement, and may have implications for the evaluation of persons with metabolic syndrome.	Purely biological approach
Adejumo, 2019 [32]	2007–2014 US	Retrospective cross-sectional study DB: HCUP-NIS *n* = 210,660 Age ≥18	ICD-9 NIS does not contain information on how NAFLD was diagnosed.	Incidence Prevalence	Sex Ethnicity SES	Larger rate of increase in NAFLD hospitalization among males vs. females (83/100,000 vs. 75/100,000), Hispanics vs. Whites vs. Blacks (107/100,000 >. 80/100,000 > 48/100,000), and government-insured or uninsured patients vs. privately insured (94/100,000 vs. 74/100,000).	Hospitalizations with NAFLD are rapidly increasing in the US, with a disproportionately higher burden among certain demographic groups: males, Hispanics, and non-privately-insured population.	The diagnostic modality is unknown. Does not perform an intersectional analysis.
Golovaty, 2020 [33]	2005–2014 US	Cross-sectional analysis DB: NHANES *n* = 2627 of low-income adults Age ≥ 20	USFLI	Prevalence	SES (Food insecurity)	29% (95% CI 26–32%) were food-insecure. NAFLD prevalence food-insecure 34% vs. NAFLD prevalence food-secure 31% (*p* = 0.21) Multivariable model: food-insecure adults are more likely to have NAFLD (adjusted OR 1.38, 95% CI 1.08-1.77)	Food insecurity may be independently associated with NAFLD among low-income adults in the US.	Does not perform an intersectional analysis.
Tutunchi, 2021 [34]	2019 Iran	Case-control study *n* = 210 (95 case vs. 115 controls) Age 20–60	Ultrasonography	Prevalence	SES (FI)	FI 56.8% in cases, with NAFLD and 26.1% in controls without NAFLD (*p* < 0.001). The likelihood of NAFLD was higher in the food-insecure (OR 2.2, 95% CI: 1.12–3.43), depressed (OR 1.9, 95% CI 1.02–3.62), overweight (OR 2.6, 95% CI 1.81–3.92), and obese (OR 2.9, 95% CI 2.02–5.34) subjects.	The prevalence of food insecurity in patients with NAFLD is significantly higher compared to controls.	Does not perform an intersectional analysis.
Hu, 2020 [35]	2015 China	Cross-sectional study Diabetes prevention program *n* = 930Age 40–76	Ultrasonic quantitative determination of fat content	Prevalence	SES Sex	In retired people, NAFLD prevalence: females (81.2%) > males (75%). As income increased, NAFLD prevalence of NAFLD increased progressively (*p* < 0.05).	The prevalence of NAFLD is related to the socioeconomic level and female gender (among retirees)	Only studies the retired population and does not perform an intersectional analysis.
Cho, 2021 [36]	2014–2018 South Korea	Cross-sectional study DB: KNHANES *n* = 5272 Age < 50 and ≥ 65	HSI and CNS	Prevalence	SES	Individuals with low SES (OR 1.70, 95% CI 1.42–2.04, *p* < 0.001) or low HGS (OR 12.16, 95% CI 9.55–15.49, *p* < 0.001) had a significantly higher risk of NAFLD. Low SES combined with a low HGS increased the risk of NAFLD when adjusted for all the covariates (OR 2.48, 95% CI 1.35–4.55, *p* = 0.003), compared with individuals with a high SES and a high HGS (OR 1).	Both low SES and low HGS are independently and synergistically associated with an increased risk of NAFLD in middle-aged Korean adults.	Does not perform an intersectional analysis.

Footnote: AAC (abdominal aortic calcification), ALT (alanine aminotransferase), AOR (adjusted odds ratio), AST (aspartate aminotransferase), BMI (body mass index), CI (confidence interval), CNS (comprehensive NAFLD score), DB (database), FI (food insecurity), HCUP (Healthcare Cost and Utilization Project), HGS (handgrip strength), HSI (hepatic steatosis index), ICD-9 (international classification of disease), KNHANES (Korean national health and nutrition examination survey), M (men), MESA (multi-ethnic study of atherosclerosis), NAFLD (non-alcoholic fatty liver disease), NIS (national inpatient survey), NHANES (national health and nutrition examination survey), OR (odds ratio), PMP (postmenopausal), RF (risk factor), SES (socioeconomic status/position), USFLI (ultrasound fatty liver index), W (women).

## Data Availability

Not applicable.
